# Racial and linguistic ideologies and formative assessment practices of U.S. science teachers: A preregistered conceptual replication and extension

**DOI:** 10.1371/journal.pone.0327859

**Published:** 2025-07-17

**Authors:** Quentin C. Sedlacek, Nickolaus Ortiz, Catherine Lemmi, Maricela León, Kimberly C. Feldman

**Affiliations:** 1 Department of Teaching & Learning, Annette Caldwell Simmons School of Education & Human Development, Southern Methodist University, Dallas, Texas, United States of America; 2 Department of Middle and Secondary Education, Georgia State University, Atlanta, Georgia, United States of America; 3 School of Education, California State University, Chico, Chico, California, United States of America; 4 Southwest Academy, Baltimore County Public Schools, Woodlawn, Maryland, United States of America; Lamar University, UNITED STATES OF AMERICA

## Abstract

Language, race, and racism are deeply connected in the United States and throughout the world. Over the past 10 years, a growing body of research has argued that many language ideologies and racial ideologies are closely associated with each other. However, few studies have sought to quantify these proposed associations by directly measuring the language ideologies, racial ideologies, and language-related behaviors of participants. In a recently published study of K-12 preservice teachers, we identified substantial correlations between racial ideologies and seemingly race-neutral language ideologies, as well as correlations between these language ideologies and the written feedback participants generated during formative assessment of student science writing. These findings have important implications for the social sciences; however, their significance is contingent in part on their reproducibility and generalizability. For this reason, we conducted a preregistered conceptual replication and extension using a larger sample of current U.S. science teachers (*n* = 387 teachers from 42 U.S. states). We demonstrate that all but one of the findings from our previous study are successfully replicated, and we identify several new findings. We discuss implications for research in science education, linguistic anthropology, social psychology, and sociology.

## Introduction

Language, race, and racism are deeply connected in the United States and throughout the world. Over the 10 years since Flores and Rosa [1] coined the term *raciolinguistic ideology*, a growing body of research has argued that many language ideologies and racial ideologies are closely associated with each other. Language ideologies are systems of beliefs about language(s) and their speaker(s) that can influence social interaction–for example, beliefs about the superiority of standardized forms of language over non-standardized forms [1–4]. Similarly, racial ideologies are systems of beliefs about the nature of race and racial identity, such as the belief that race is a “natural” category grounded in biology or the belief that race is an arbitrary category constructed by human societies [5,6]. Research exploring associations between racial ideologies and language ideologies has largely been qualitative in nature [7], although its key tenets are supported by past quantitative research (e.g., [8]). A small but growing body of quantitative research has sought to empirically explore raciolinguistic phenomena such as racially biased perceptions of language, usually spoken language (e.g., [8–10]). However, these studies typically do not directly measure the language ideologies and racial ideologies believed to be associated with such biases, instead typically measuring biased behavioral responses only.

Quantifying relationships between language ideology, racial ideology, and behavior could have important implications for multiple fields of social science. Language ideologies have been the subject of extensive qualitative (and some quantitative) research in fields such as sociolinguistics and linguistic anthropology [11], while racial ideologies and racially biased behaviors have been the subject of extensive qualitative and quantitative research in social psychology [12–14] and sociology [15–17]. Combining these fields of research using raciolinguistic ideology theory could prove highly generative. While qualitative research has convincingly demonstrated the existence and character of raciolinguistic ideologies, quantitative work could further our understanding of the scope and scale of these ideologies and the inequities they perpetuate. In doing so, quantitative work on raciolinguistic ideologies could help inform strategies for researchers, policymakers, and practitioners to address these ideologies, and could help develop tools for assessing the effects of specific pedagogical or policy interventions.

In a recently published study [18], our team began to address this gap in the specific context of U.S. K-12 science education, using quantitative surveys and performance tasks to investigate ideology and behavior among a population of K-12 preservice teachers. Abundant research suggests that seemingly race-neutral beliefs often mask covert forms of racism, as shown in a meta-analysis by Yi et al. [12], and our work similarly found substantial correlations between problematic racial ideologies and seemingly race-neutral prescriptive beliefs about science writing [18]. We also found associations between these ideologies and the written feedback participants generated during formative assessment of student science writing. Our findings go beyond previous quantitative raciolinguistics research that has measured accentedness judgments (e.g., [8,9]) or evaluative responses to language (e.g., [10]), and provides one of the first quantitative tests of the premise that behavioral responses to language are related to both language ideology and racial ideology. Our work thus has significant implications for sociolinguistics, linguistic anthropology, social psychology, and sociology, as well as science education and teacher education.

The significance of these findings is contingent in part on the idea that they are reproducible and generalizable to broader populations, such as in-service science teachers or American adults more generally. Many scholars believe science suffers from a reproducibility crisis [19], and the generalizability of our previous study was constrained by its small sample (65 pre-service teachers of science and other subjects from 2 college campuses). To better establish the reproducibility and generalizability of our findings, we sought to conceptually replicate and extend these findings in a larger preregistered study of current U.S. science teachers *(n =* 387 teachers from 42 U.S. states).

### Ideology and writing in science education

Language practices associated with racially marginalized communities, such as African American Language (AAL), are often heavily stigmatized in classrooms and other social contexts [1,20,21]. There is a broad consensus among linguists that such stigma is not grounded in any empirical research on language and instead is simply an ideology driven by historical racism [20,21]. As early as the 1970s, national professional organizations devoted to language arts education issued statements affirming students’ right to use and maintain their language practices [22,23], and many linguists and educators assert that AAL is a valuable asset that can support students’ language learning, content learning, and/or identity development in schools [18,20,22–27]. Despite this longstanding scholarly consensus, U.S. schools often implicitly or explicitly prohibit using AAL in classrooms, curtailing students’ opportunities for sensemaking [20,23,24]. These prohibitions are harmful and counterproductive; research has shown that more expansive approaches to language, in which students are allowed or encouraged to draw on multiple aspects of their linguistic repertoire, may serve valuable academic and social purposes in the classroom, including for science and mathematics learning [28–32].

While teachers’ language ideologies and responses to AAL have frequently been researched in language arts education (e.g., [27,33]), they have rarely been studied in other K-12 content areas such as STEM education [4,18,34]. However, language ideologies represent critical and necessary areas of focus even within these other content areas. For example, many science educators believe students should use formal scientific language in class, sometimes at the exclusion of other varieties of language [18,35], despite research showing that students benefit from opportunities to paraphrase or translate scientific ideas into their own words [30,36,37]. We refer to this notion–that students should exclusively or almost exclusively use relatively formal scientific language when writing or speaking in science courses–as a “language-exclusive ideology” [4], encompassing both prescriptive beliefs about language and restrictive pedagogical practices or policies aligned with those beliefs. Studying language-exclusive ideologies is important because these ideologies might impede the use of AAL as an asset for science learning by driving teachers to criticize or prohibit speaking or writing using AAL, shutting down valuable opportunities for student sensemaking and identity development [18].

Language-exclusive ideology might do more than simply prescribe the way teachers believe students ought to write or speak; it may also influence how teachers themselves read and listen to students’ language use. In doing so, it may interfere with formative assessment in science and other content areas. In a recent mixed-methods study of 65 preservice teachers of science and other subjects [18], we found that participants who expressed a stronger language-exclusive ideology in a survey appeared more prone to making questionable inferences about students’ scientific understanding based on purported samples of student writing during a formative assessment performance task. Participants who expressed stronger language-exclusive ideology also tended to write shorter feedback on all student science writing samples, despite often professing to believe that feedback on writing is essential to support student learning. Although this association does not necessarily imply a causal relationship, it suggests the possibility that language-exclusive ideologies might function in ways that limit the amount of useful feedback students receive on their science writing. Finally, these same language-exclusive participants appeared prone to giving feedback in racially biased ways–often “correcting” students’ use of a valid and systematic grammatical feature associated with African American Language as though it were an “error,” yet often overlooking “errors” that were racially unmarked, i.e., deviations from standard spelling, grammar, or punctuation that are not specifically associated with African American Language in U.S. society. These patterns appeared to reflect differences in ideology rather than differences in pedagogical content knowledge. We found that our survey scale measuring language-exclusive ideology was a stronger predictor of these patterns compared with a previously validated scale measuring science teachers’ knowledge of language as an epistemic tool for teaching science [18,35].

We also found that seemingly race-neutral beliefs about science writing pedagogy measured through our language-exclusive ideology scale (e.g., the belief that students should avoid using personal pronouns in science writing) were, in fact, substantially correlated with teachers’ racial ideologies and their asset-based attitudes (or lack thereof) towards African American Language. Teachers with stronger language-exclusive ideology tended to express stronger agreement with racial essentialism, the ideology that race is a biologically real and meaningful category rather than a social construct. Teachers with stronger language-exclusive ideology were also less likely to view African American Language as a valid form of language and a potential asset in STEM subjects such as science or mathematics. Such correlations between language ideologies and racial ideologies have been widely theorized and qualitatively studied in the decade since Flores and Rosa [1] coined the term “raciolinguistic ideology.” However, the relationships between language ideologies and racial ideologies have rarely been studied quantitatively.

### Motivation and research questions

These findings may have important implications for teacher education, linguistic anthropology, social psychology, and sociology. However, their significance is in part contingent on the idea that these findings are reproducible and generalizable to broader populations of science teachers, teachers of other subjects, or American adults more generally. Many scholars believe science suffers from a reproducibility crisis [19], and the generalizability of our previous study was limited by its relatively small and heterogeneous sample of 65 pre-service teachers of science and other subjects. To better establish the reproducibility and generalizability of our findings, we sought to conceptually replicate and extend them in a larger preregistered study of current U.S. science teachers (*n *= 387). We pre-registered our study by posting our research design, sampling plan, instrumentation, and a detailed analysis plan through the Open Science Framework; pre-registering an analysis plan strengthens confidence in research findings by minimizing the risk of “p-hacking” and publication bias [38].

The current study is a *conceptual* replication, rather than an *exact* replication, because we used the same instrumentation and variables of interest as our original study [18] but replaced two covariates from our original study with covariates more appropriate for a larger sample of in-service (rather than pre-service) teachers. The original study contained a binary variable denoting whether pre-service teachers expected to primarily teach science or a different, non-science subject; our conceptual replication replaces this with a binary variable denoting whether in-service teachers *actually teach* biology, life science, or general science courses (i.e., the science courses most relevant to the study materials and performance task) or only teach other science courses such as physics or chemistry. The original study also contained a binary variable denoting which university a pre-service teacher attends (only two campuses were included in the original study); our conceptual replication replaces this with an integer variable that denotes the grade level most commonly taught by a participating in-service teacher.

We examined eighteen distinct preregistered hypotheses, which can be summarized by the following research questions:

RQ1. Are science teachers’ language-exclusive ideologies, racial essentialism, and asset-based attitudes toward African American Language significantly correlated with each other? (Hypotheses 2–4)

RQ2. Are science teachers’ language-exclusive ideologies associated with their self-reported grading practices (Hypothesis 1) and their behavior during a formative assessment performance task (e.g., grading science writing samples, writing feedback to students, writing inferences about student understanding) after controlling for the science discipline(s) they teach, the grade level of students they teach, and their knowledge of language as an epistemic tool for teaching science? (Hypotheses 5–18)

### Positionality

In terms of our positionalities, various members of our team identify as White, Black, or Hispanic; as women or men; as cisgender and straight or LGBTQ; and as mother tongue speakers of African American Language or Black Language, English, and/or Spanish. Importantly, we view positionality statements not merely as a tool for informing readers about our identities but also as an avenue for critiquing and moving beyond the traditionally extractive and oppressive character of much social science research [39,40]. In this study, we were motivated by the Charity Hudley Rule for Liberatory Linguistics [41], which argues researchers studying marginalized communities and their language practices should explicitly describe efforts to “increase the participation of [insider] community members at your university, in your department, and in your research area” (p. 136). Some of our efforts include organizing and funding guest lectures by scholars who research African American Language from insider perspectives and citing and centering such scholars in our scholarship. We have also sought to support writing and publishing by African American community members inside and outside academia by offering research participants in some of our studies a choice among several forms of compensation, including a gift card to an online bookseller that centers African American writers and perspectives.

## Materials and methods

We measured teachers’ formative assessment practices using a short performance task and measured ideologies, attitudes, and knowledge using a short survey. This study was approved by the Southern Methodist University Institutional Review Board under protocol 24–043. All participants provided informed written consent through the Qualtrics survey. The combined performance task and survey instrument are available online as part of our preregistration protocol (osf.io/fjwbv). To strengthen the design of our preregistration, we reviewed Bakker et al. [42] for a list of 29 elements of research design which, when left unspecified in preregistration plans, can act as “degrees of freedom” that result in low-quality or underspecified preregistrations. Each of these 29 elements was addressed within our preregistration protocol, and any deviations from the protocol are explicitly noted.

### Sampling

By reviewing publicly available school-, district-, and state-level websites and databases, we curated a list of 9,031 email addresses for middle school or high school science teachers spread across all 50 U.S. states. Approximately 13% of these email addresses came from a single state that maintains a large publicly accessible database of teacher contact information; no other individual state represented more than 10% of the email corpus. Another ~29% of the emails came from four very populous states, all of which were among the six most populous states in the U.S. Another ~29% came from ten additional states of varying sizes. The remaining ~29% came from the remaining 35 states. The corpus included email addresses from at least three different schools or districts in every state.

We recruited participants via email using a Qualtrics mailmerge, meaning each prospective participant received an individualized survey link. This enabled us to verify participants’ identities and prevent duplicate submissions or submissions from spambots. We sent email invitations in April and May of 2024 and continued recruiting participants until after we had surpassed 377 responses, the minimum required based on a power calculation described in our preregistration protocol. We ultimately had 387 completed responses, a response rate of 4.3%. Participants taught in 42 of the 48 contiguous lower U.S. states (no responses were received from Alaska or Hawai‘i). All participants received an Amazon.com gift card after completing the study.

As shown in Table 1, racial and gender demographics of our sample were broadly similar to those of science teachers in the United States, of whom approximately 88% identify as White, 5% as Black, and 7% as Hispanic [43] and of whom approximately 60.5% identify as female and 39.5% as male [44].

**Table 1:** Participant Demographics.

	*N*	%
**Science course(s) taught (non-exclusive; may total more than 100%)**		
Biology and/or other Life Science	196	50.6
Chemistry, Physics, and/or other Physical Science	192	49.6
General Science	78	20.2
Another type of science	58	15.0
**Grade level most commonly taught**		
6th	17	4.4
7th	22	5.7
8th	29	7.5
9th	87	22.5
10th	98	25.3
11th	100	25.8
12th	34	8.8
**Racial identity (non-exclusive; may total more than 100%)**		
White	316	81.7
Black or African American	14	3.6
Hispanic, Latina, Latino, or Latinx	13	3.4
Southeast Asian	12	3.1
East Asian	10	2.6
American Indian or Alaska Native	9	2.3
Multiracial	9	2.3
South Asian	4	1.0
Middle Eastern	3	0.8
Native Hawaiian or Pacific Islander	2	0.5
Other	16	4.1
Prefer not to say	16	4.1
**Gender identity**		
Female	240	62.0
Male	128	33.1
Non-binary, third gender, more than one gender, or prefer not to say	19	4.9
**Multilingual**		
Yes	77	19.9
No	310	80.1
**Currently interacts (or previously interacted) regularly with AAL users**		
Yes	141	36.4
Maybe/ not sure	42	10.9
No	204	52.7
**Dialect region** ^a^		
New England	105	27.1
Midland/North	139	35.9
South	78	20.2
West	65	16.8

^a^Based upon approximate boundaries of dialect regions of the United States as defined by Labov [45]. For purposes of this study, New England includes the 6 states north and/or east of Connecticut; South includes the 13 states south and/or east of Virginia, West Virginia, Kentucky, Arkansas, and Texas; West includes the 11 contiguous states west of Montana, Wyoming, Colorado, and New Mexico; Midland/North includes the remaining 18 contiguous states.

The language backgrounds of our sample also appeared similar to those of the general U.S. population, approximately 22% of whom use a language other than English at home [46]. However, we did not gather data on the exact languages used by participants or the contexts in which they use these languages.

### Survey measures

We measured language-exclusive ideologies using a five-item Likert-type scale (Cronbach’s alpha = 0.752) developed by the authors, which exhibited concurrent validity with both self-reported formative assessment practices and performance task outcomes of preservice teachers in a prior study [18]. This scale measured agreement or disagreement with items such as “Students need to be fairly skilled writers before writing activities will be useful in a science class” and “If a student does not know the scientific vocabulary terms to describe a concept, they do not yet understand the concept.” We argue that agreement with such items conveys a broader prescriptive belief that there is only one valid way (or a narrow range of valid ways) to convey scientific ideas in writing. None of the items on this scale included any reference to race, ethnicity, or racism.

We measured racial essentialism using a twenty-two item Likert-type scale (Cronbach’s alpha = 0.869) developed and validated by Williams and Eberhardt [47]; this instrument has previously been used to study racial ideologies in science education contexts (e.g., [48]). This scale measured agreement or disagreement with items such as “Racial groups are primarily determined by biology” and “It’s natural to notice the racial group to which people belong.” These items were originally designed to measure whether respondents believe race is “biological…natural…easily discernible…stable across time…stable across contexts…and stable within individuals” [47]. We argue that agreement with these items reflects a set of widely debunked and harmful notions about race [5,48].

We measured teachers’ asset-based attitudes toward African American Language using eight modified items from a Likert-type scale (Cronbach’s alpha = 0.793) developed by Blake and Cutler [27] and also used in our prior study [18]. This scale measured agreement or disagreement with items such as “African American English (sometimes called AAE, AAVE, or Ebonics) is a form of English” and “It is educationally sound to use a student’s first language or dialect as a way of teaching that student the standard language of a community,” and also measured agreement or disagreement with reverse-coded items such as “Standard English is dominant in schools and business because it is the best form of English” and “It is inappropriate for teachers or students to use AAE in school subjects such as science or math.” We argue these items measure the degree to which respondents value linguistic diversity in general and African American Language in particular as an asset, both in STEM classrooms and in other environments.

We measured teachers’ knowledge of language as an epistemic tool for teaching science using a fifteen-item Likert-type scale (Cronbach’s alpha = 0.675) developed and validated by Fulmer et al. [35]. This scale measured agreement or disagreement with items such as “Writing to different audiences helps students to deepen conceptual understanding” and with reverse-coded items such as “As students get older, science class should not encourage students to use everyday language for science concepts.” These items were designed to measure teachers’ knowledge of language as it relates to teaching and to strategies for facilitating students’ development of conceptual understanding [35].

We measured teachers’ self-reported formative assessment practices with a five-item Likert-type scale (Cronbach’s alpha = 0.856), asking how frequently they believe they should grade students’ spelling, grammar, punctuation, use of scientific vocabulary, and use of passive voice in the context of evaluating student science writing.

Tavakol and Dennick [49] report that researchers typically accept values of alpha ranging between 0.70 and 0.95; lower values may reflect items that are not sufficiently interrelated to measure a single construct, while higher values may reflect redundancy. In our study, all scales had alphas between 0.752 and 0.869, except for the scale measuring teachers’ knowledge of language as an epistemic tool for teaching science [35], which exhibited an alpha of 0.675. This scale’s reliability was problematically low, though it was higher than sometimes exhibited by this scale in prior research (e.g., [50]). Readers should keep this issue in mind when interpreting our analyses.

### Formative assessment performance task

We also measured teachers’ formative assessment practices using a performance task which asked participants to respond to three samples of purported student writing that describe mitosis. (All three samples were actually composed by teachers and then edited by the researchers; see Sedlacek et al. [18] for details and see preregistration materials at osf.io/fjwbv for the writing samples themselves). Writing Sample #1 was short (22 words) and used many technical scientific vocabulary terms, similar to a description students might copy out of a textbook. Writing Sample #2 was longer (54 words) and used fewer technical vocabulary terms; it also included a well-researched grammatical feature known as habitual *be*, which denotes an action or process habitually repeated over a long period of time (in this case, “Cells always be dividing to heal cuts, grow, and reproduce…”). Habitual *be* is often perceived as a grammatical error, yet it is in fact a well-documented and grammatically valid feature associated with African American Language [51]. Writing Sample #3 was longer still (110 words) and included few technical vocabulary terms and no instances of habitual *be*. Instead, it included several mechanical errors that are not associated with African American Language: two misspellings, one of which was repeated twice; a missing comma prior to an appositive, which can be interpreted as either a punctuation error or grammatical error; and multiple uses of sentence-initial “so,” which sometimes elicits criticism from prescriptivists as grammatically undesirable despite its increasing usage in English [52,53]. All three writing samples conveyed accurate information about mitosis. All are the same writing samples used in Sedlacek et al. [18] and are also available in the preregistration materials.

Participants were asked to assign a grade to each writing sample (on an eleven-point scale from 0 to 10) and to quantify the level of student understanding they could infer from each writing sample (on an eleven-point scale from 0 to 10). In two open-ended items, participants were also asked first to compose feedback to the author of each writing sample, and then to write any inferences they could make about student understanding based on each writing sample.

Following our preregistered protocol, the outcomes we examined in this analysis included: the grade teachers assigned to each writing sample; the level of student understanding teachers based on each sample; the length of feedback and length of inferences (measured in characters) teachers wrote in responses to each sample; and a binary outcome measure denoting whether or not each teacher included mechanics-focused comments in their feedback (e.g., whether a teacher wrote that a student author needed to change their grammar, spelling, punctuation, etc.).

### Analysis

Following our preregistered protocol, we tested eighteen distinct hypotheses using correlation coefficients, multiple linear regression, and logistic regression.

### Tests of model assumptions

Following our preregistered protocol, we initially tested our data for possible violations of regression assumptions. Collinearity diagnostics showed that all four predictor variables included in our models exhibited variance inflation factors (VIFs) below 1.2, well within acceptable ranges. We initially tested for normality using Shapiro-Wilk tests. These indicated that *none* of the variables of interest were normally distributed except for the racial essentialism scale; in retrospect, however, we recognized that the Shapiro-Wilk test is not necessarily appropriate for Likert-type data, particularly with large sample sizes [54]. Thus, in a minor deviation from our preregistration protocol, we also visually inspected Q-Q plots; these suggested that *all* variables of interest were approximately normally distributed, except for the six variables quantifying the length of feedback and the length of inferences written by participants, all of which appeared to exhibit negative skew. Thus, we retained all variables in their original forms for analysis except for the six variables describing the length of feedback and inferences, which were transformed using a square root function that successfully addressed the normality problem. All analyses involving these variables were conducted twice, once using the transformed variables and once using the untransformed variables. All coefficients or odds ratios that were statistically significant following a Benjamini-Hochberg correction on analyses of the untransformed variables were also significant following a Benjamini-Hochberg correction [55] on analyses of the transformed variables. (One additional effect displayed statistical significance in the analysis of transformed variables but not untransformed variables: an effect of teacher grade level on the length of feedback written on Writing Sample #2. This effect is denoted with a + in the relevant table.) We report the analyses of untransformed variables here for readers’ convenience.

Also, following our preregistration protocol, we used a Breusch-Pagan test that identified heteroskedasticity in two of the linear regression models: the model predicting the grade assigned to Writing Sample #2, and the model predicting the frequency with which participants believed they should grade mechanics and vocabulary in student writing. However, neither of these dependent variables exhibited outliers, which our preregistration defined as data points with |Z| > 3.5. For these reasons, following our preregistration protocol, we used weighted least squares regression for these two models.

### Combining qualitative and quantitative data

We used quantitizing mixed methods [56] to code the qualitative feedback teachers had written in response to each of the three writing samples and integrate these codes into the quantitative dataset. We used a binary variable to denote whether feedback included comments focused on the mechanics of student writing (e.g., spelling, grammar, punctuation, sentence structure), excluding comments about scientific vocabulary. First, the lead authors and two coders briefly discussed the “mechanics” code included in the preregistration protocol and discussed how this code might be applied to approximately 15 samples of feedback teachers had written in our prior study [18]. Second, the lead author separated the formative assessment performance task data in the *current* study from the rest of the dataset (so that coders would not know which responses were written by teachers with higher or lower levels of language-exclusive ideology). Next, the lead author reviewed all responses and determined that none of the 1,161 individual pieces of feedback needed to be excluded based on preregistration criteria (specifically, none of the feedback consisted of text strings which were both irrelevant to the study and longer than 5 characters in length). Then, following our preregistration protocol, the two coders independently applied the “mechanics” code to all 1,161 pieces of feedback. The lead author checked intercoder agreement, which was 96.0% across all responses; Cohen’s kappa was 0.835. Although our intercoder agreement was 96.0% across all responses, it was only 75.1% among the small subset of responses that were coded Mechanics = 1 by one or both raters.

Our preregistration protocol stated that if we failed to achieve at least 80% simple agreement across the subset of responses coded Mechanics = 1 by one or both raters, we would have coders meet to discuss the coding scheme, revise the code, and then assign two new coders to re-code the entire dataset using this new code. However, we recognized at this stage that a measure of reliability across the full dataset would be more appropriate, and that Cohen’s kappa is a more appropriate measure of reliability than simple agreement given the relatively low proportion of responses that received the mechanics feedback code (between 13% and 15%, depending on the coder). Cohen’s kappa was 0.835 for the full dataset, a value considered to be high [57]. Thus, in a deviation from our preregistered protocol, we did not revise the code definition and re-code the data. Instead, we continued with the next preregistered stage of our analysis.

The two coders met to discuss and resolve disagreements, and the coded values were reintegrated into the dataset as a binary variable (1 = feedback included one or more comments about writing mechanics such as spelling, grammar, punctuation, or sentence structure; 0 = feedback did not include comments about writing mechanics). Finally, we conducted a logistic regression of the form-focused feedback variable on language-exclusive ideology, controlling for the science discipline(s) participants teach, the grade level of students they teach, and their knowledge of language as an epistemic tool for teaching science.

### Correction for multiple comparisons

We corrected for multiple tests of statistical significance using a Benjamini-Hochberg correction [55] calibrated for 63 tests of statistical significance. This enabled us to test our eighteen hypotheses while also looking for additional potentially meaningful findings among all coefficients in the linear and logistic regression models. Because the Benjamini-Hochberg correction controls the False Discovery Rate rather than the Familywise Error Rate, it is inappropriate to report adjusted *p*-values for individual statistics; instead, we simply indicate whether each individual statistic was significant following the Benjamini-Hochberg procedure with the False Discovery rate set to.05.

## Results

### Relationships among language ideology, racial ideology, and attitudes toward African American Language

In our preregistration, we hypothesized that measures of language-exclusive ideology, racial essentialism, and asset-based attitudes towards African American Language would all have significant correlations. Ultimately, the Pearson correlation between language-exclusive ideology and racial essentialism was *r *= 0.366, the Pearson correlation between racial essentialism and asset-based attitudes towards African American Language was *r = *−0.514, and the Pearson correlation between asset-based attitudes towards African American Language and language-exclusive ideology was *r = *−0.409. All three correlations were significant following a Benjamini-Hochberg correction.

### Formative assessment practices

Table 2 shows that most of our preregistered hypotheses related to quantitative formative assessment practices were borne out. The language-exclusive ideology measure was positively related to the frequency at which teachers self-reported grading the mechanics and vocabulary of student writing (*β *= 0.546), positively related to the level of student understanding they inferred from Writing Sample #1 (*β *= 0.236), and negatively related to both the grade teachers gave (*β *= −0.161) and the level of student understanding they inferred (*β *= −0.147) based on Writing Sample #2, and was negatively related to both the grade teachers gave (*β *= −0.151) and level of student understanding they inferred (*β *= −0.255) based on Writing Sample #3. All of these relationships were statistically significant after controlling for the science discipline(s) and grade level(s) participants teach and their knowledge of language as an epistemic tool for teaching science. Several of these relationships (e.g., lower grades and lower levels of inferred understanding based on Writing Sample #2) appeared in our previous smaller study but did not exhibit statistical significance, perhaps due to insufficient statistical power.

**Table 2:** Standardized Coefficients from Multiple Linear Regression^a^ of Grades and Inferences of Student Understanding on Predictor Variables.

Variable	Frequency of grading mechanics and vocabulary^a^	Grade assigned to the writing sample			Understanding inferred from the writing sample		
		#1	#2^a^	#3	#1	#2	#3
**Language-Exclusive Ideology**	0.546* (0.043)	0.102 (0.051)	−0.161* (0.051)	−0.151* (0.051)	0.236* (0.050)	−0.147* (0.051)	−0.255* (0.050)
**Teaches Life Science or General Science Course(s)**	−0.052 (0.044)	−0.019 (0.053)	−0.040 (0.053)	−0.085 (0.053)	0.088 (0.052)	0.018 (0.053)	−0.114 (0.051)
**Grade Level of Students**	0.008 (0.045)	−0.093 (0.053)	0.004 (0.053)	0.020 (0.053)	−0.051 (0.052)	0.031 (0.053)	0.075 (0.051)
**Knowledge of Language as an Epistemic Tool for Teaching Science**	0.022 (0.043)	0.017 (0.051)	0.003 (0.051)	0.023 (0.051)	−0.075 (0.050)	0.019 (0.051)	0.017 (0.049)

*Note.* * denotes coefficients that were statistically significant following a Benjamini-Hochberg correction with the False Discovery Rate set at.05.

^a^Due to heteroskedastic errors in the models predicting the frequency participants believe they should grade mechanics and the actual grades they gave on Writing Sample #2, weighted least squares regression was used for these models.

As shown in Table 3, teachers who expressed higher levels of language-exclusive ideology wrote shorter feedback and generated shorter inferences in response to all three writing samples. We also found that teachers of life science or general science courses (who may be more likely to teach mitosis content) tended to write longer feedback (*β *= 0.160) and longer inferences (*β *= 0.187) on Writing Sample #1 and longer feedback (*β *= 0.132) and longer inferences (*β *= 0.146) on Writing Sample #2, but not on Writing Sample #3, and that teachers of older students (who may expect their students to write more complex texts) tended to write longer feedback on Writing Sample #1 (*β *= 0.141) and Writing Sample #3 (*β *= 0.128), and tended to generate longer inferences in response to Writing Sample #1 (*β *= 0.147) and Writing Sample #2 (*β *= 0.140).

**Table 3:** Standardized Coefficients from Multiple Linear Regression of Length of Feedback and Inferences about Student Understanding on Predictor Variables.

Variable	Length of Feedback on Writing Sample			Length of Inferences made from Writing Sample		
	#1	#2	#3	#1	#2	#3
**Language-Exclusive Ideology**	−0.255* (0.050)	−0.187* (0.051)	−0.230* (0.050)	−0.183* (0.050)	−0.229* (0.050)	−0.253* (0.050)
**Teaches Life Science or General Science Course(s)**	0.160* (0.051)	0.132* (0.052)	0.079 (0.052)	0.187* (0.052)	0.146* (0.052)	0.042 (0.052)
**Grade Level of Students**	0.141* (0.051)	0.119^+^ (0.052)	0.128* (0.052)	0.147* (0.052)	0.140* (0.052)	0.041 (0.052)
**Knowledge of Language as an Epistemic Tool for Teaching Science**	0.042 (0.050)	0.037 (0.050)	0.053 (0.050)	0.020 (0.050)	−0.020 (0.049)	0.035 (0.050)

*Note.* * denotes coefficients that were statistically significant following a Benjamini-Hochberg correction with the False Discovery Rate set at.05.

+ denotes a finding that exhibited statistical significance in the analysis of transformed variables but not untransformed variables.

In our preregistration protocol, we also hypothesized that science teachers’ language-exclusive ideologies would be associated with the likelihood that they included mechanics-focused feedback in their responses to Writing Samples #2 and #3 after controlling for other variables. (In our previous study, language-exclusive ideology was positively related to the use of mechanics-focused feedback in response to Writing Sample #2 but not Writing Sample #3.)

Table 4 shows that language-exclusive ideology was *not* significantly related to whether or not science teachers gave mechanics-focused feedback on either Writing Sample #2 (Odds Ratio 1.033, 95% CI {0.991, 1.077}) or Writing Sample #3 (Odds Ratio 1.042, 95% CI {0.996, 1.090}) after controlling for other variables. Thus, our previous finding that the language-exclusive ideology scale was associated with biased responses to AAL features was not replicated.

**Table 4:** Binary Logistic Regression of Mechanics-Focused Feedback Practices Observed in Performance Task on Predictor Variables.

Variable	Gave mechanics-focused feedback on Writing Sample #2				Gave mechanics-focused feedback on Writing Sample #3			
	B (SE)	Wald	Odds Ratio Exp(B)	95% CI for Odds Ratio	B (SE)	Wald	Odds Ratio Exp(B)	95% CI for Odds Ratio
**Language-Exclusive Ideology**	0.033 (0.021)	2.340	1.033	(0.991, 1.077)	0.041 (0.023)	3.258	1.042	(0.996, 1.090)
**Teaches Life Science or General Science Course(s)**	−0.028 (0.263)	0.012	0.972	(0.580, 1.628)	−0.120 (0.278)	0.186	0.887	(0.514, 1.530)
**Grade Level of Students**	−0.067 (0.082)	0.662	0.935	(0.796, 1.099)	0.033 (0.090)	0.136	1.034	(0.867, 1.233)
**Knowledge of Language as an Epistemic Tool for Teaching Science**	−0.017 (0.020)	0.657	0.984	(0.945, 1.024)	0.027 (0.022)	1.435	1.027	(0.983, 1.073)

We noted that 24% of respondents gave mechanics-focused feedback on a writing sample with a single AAL feature, while only 20% gave mechanics-focused feedback on the writing sample with deviations from standard spelling, grammar, or punctuation that were not specifically associated with African American Language. In an additional exploratory, non-preregistered analysis, an asymptotic McNemar’s test [58] determined that there was not a statistically significant difference in the proportion of respondents giving mechanics-focused feedback on Writing Samples #2 and #3, *p* = .098 (not included in our Benjamini-Hochberg correction).

## Discussion

Collectively, these findings have important implications for multiple fields. In sociolinguistics and linguistic anthropology, our findings support arguments by Flores and Rosa [1], Martin et al. [59], and others that suggest attempts to contest oppressive *language* ideologies must attend to issues of *racial* ideology as well, because these ideologies are often deeply interconnected. While theories and methods from linguistic anthropology are used extensively in research on first language and second language education, they are more rarely invoked in science education or in STEM education more generally (with the notable exception of systemic functional linguistics, e.g., [60–62]). STEM education would benefit from further application of linguistic anthropological insights on language *ideologies*, particularly raciolinguistic ideologies; for initial examples of this work, see Sedlacek et al. [18], Lemmi et al. [4], and Harper and Kayumova [63].

In social psychology, our findings advance researchers’ ongoing agenda to understand how essentialism is related to other psychological constructs and behaviors [64,65]. The link we have shown between racial essentialism, prescriptivist beliefs about language, and behavioral responses to language may suggest a new line of inquiry for studying racial stereotyping and prejudice, both in STEM education and beyond. For example, do teacher education programs designed to disrupt racial essentialism among biology teachers and biology students (e.g., [66,67]) indirectly lead to changes in language ideologies, as well? Could language ideologies somehow moderate or mediate processes of ideological change related to racial essentialism? Collaborative investigations of such questions, combining methods and expertise from science education and social psychology, could help psychologists understand the deeper structure of essentialist beliefs while also extending science educators’ understanding of strategies to disrupt essentialism in biology education.

In sociology, our findings suggest that beliefs about language prescriptivism may function as a previously under-researched form of color-evasive ideology, whereby oppressive beliefs about race are covertly embedded in seemingly race-neutral discourses and beliefs [68,69]. Color-evasive ideologies remain common among U.S. STEM educators at the college level [70] and evidence suggests these ideologies might impede educators’ adoption of effective, inclusive teaching practices [71]. Understanding color-evasive ideologies in greater detail—especially those that directly relate to STEM education, such as the language-exclusive ideologies measured in our study—could generate insights that might inform future efforts to implement effective, inclusive teaching practices in STEM education.

In STEM education, our findings suggest that language-exclusive ideology may be an impediment to effective formative assessment in science and other disciplines. Teachers of science (and perhaps other content areas as well) appear to exhibit better formative assessment practices if they also espouse more language-*inclusive* ideology—that is, if they value multiple varieties of language in the classroom, including African American Language. If the observed relationships between language ideology and formative assessment turn out to be causal, then promoting language-inclusive ideology in science teacher education might lead teachers to generate more extensive feedback for students and might lead them to devote additional time or effort to making well-justified inferences about student understanding. It is also possible that promoting language-inclusive ideology may lead teachers of science, mathematics, and other subjects to provide students with more substantive, content-focused feedback on their writing. In both our original study and this conceptual replication, participants with lower levels of language-exclusive ideology reported grading the mechanics and vocabulary of student writing less frequently and tended to provide lengthier feedback and lengthier inferences about student understanding compared with their more language-exclusive colleagues. Our findings echo those of earlier work suggesting that ideologies about language in general, and AAL in particular, may shape the ways that teachers select and implement effective or high quality pedagogical practices such as providing students with feedback [72].

However, one finding did fail to replicate: in our original study, more language-exclusive teachers were also more likely to critique habitual *be* in student writing, yet in the replication, both more- and less-language-exclusive teachers appeared to critique habitual *be* at comparable rates. While teachers with more language-*inclusive* ideologies might be expected to critique this AAL feature at lower rates, our replication data suggests otherwise. Interestingly, this pattern was not clearly attributable to the behavior of either language-exclusive or language-inclusive teachers; language-inclusive teachers critiqued habitual *be* at substantially higher rates in the replication than in our original study, while language-exclusive teachers critiqued habitual *be* at substantially lower rates in the replication than in our original study. This may indicate that our initial finding of a correlation between language-exclusive ideology and anti-AAL bias in feedback was spurious, and disappeared in the replication due to simple regression to the mean. (To be clear, we do find evidence of anti-AAL bias in both studies–in both studies, an AAL feature which is not an error is “corrected” by a substantial fraction of participants–but this bias was uncorrelated with language ideology in the replication.)

Alternatively, this failed replication may indicate that the relationship between language-exclusive ideology and biased patterns of feedback differs between a population of pre-service teachers of various subjects and a population of in-service teachers of science only, or between teachers with different amounts of teaching experience. The latter possibility is supported by the fact that language-exclusive ideology was correlated with racial essentialism in both studies; perhaps racial essentialism was associated with biased responses to AAL, but this bias is less severe among more experienced teachers. A study of gender bias in science assessment practices in E.U. countries found that such bias tended to be stronger or more common among early-career teachers rather than experienced teachers [73]. While we did not collect data on participants’ years of teaching experience in our replication, our sampling of email addresses from publicly available websites and databases was likely to preferentially include teachers with at least one and likely multiple years of experience (since these teachers have been at their current workplaces long enough to be added to school websites).

Further research should explore these phenomena using causal methods. These might include experiments that control for all features of student writing except for the presence or absence of AAL features, or tests of interventions that seek to change science teachers’ language ideologies and measure change(s) in teachers’ formative assessment practices. Additional qualitative and mixed-methods research should also explore *how* science teachers make sense of information about African American Language and apply such knowledge in their classrooms.

### Limitations

Our methods had important limitations. For example, our sampling procedure relied on the public availability of teacher contact information, meaning it preferentially identified science teachers who (1) are employed by schools large enough to offer lists of teacher contact information on their websites and (2) have been in their jobs long enough to be listed on these websites. These may introduce important biases into the results; for example, as discussed above, the overrepresentation of teachers in larger schools and teachers with multiple years of experience (compared with teachers in smaller schools and first-year teachers) may have shaped our findings in important ways. Our instruments also had limitations—for example, the low reliability of the scale measuring teachers’ knowledge of language as an epistemic tool, which may have contributed to the lack of observed relationships between this construct and other constructs measured in our survey.

Given our goal of conceptually replicating a previous correlational study, this time with a larger and more externally valid sample, we did not conduct a controlled experiment (though our performance task could easily be adapted into an experiment in future studies–for example, by varying which of the three writing samples contained an instance of habitual *be,* or by varying the verb tenses in each of writing samples #1, #2, and #3). With this in mind, we cannot make causal claims about the relationship between language-exclusive ideology and formative assessment practices. We can merely point to correlational findings and argue that causal claims about these relationships are *plausible* based on convergent evidence from other fields. Further research is needed to explore relationships among language ideologies, racial ideologies, and classroom formative assessment practices in greater depth, both in science education and other fields.

## Conclusions

In this conceptual replication, we reproduce our previous finding that seemingly race-neutral beliefs about writing pedagogy are in fact correlated with essentialist ideologies of race. We also reproduce and extend our previous findings about the formative assessment practices of teachers with more- or less-prescriptive beliefs about student writing, showing that teachers with relatively language-inclusive ideology appear to respond differently to student writing samples compared with their more language-exclusive colleagues.

Our findings provide quantitative evidence in support of raciolinguistics, a set of linguistic anthropological theories which postulate connections between racial ideology and seemingly race-neutral beliefs about language. These findings suggest that efforts to improve formative assessment in science education should address not only teachers’ knowledge of language, but their language ideologies and racial ideologies as well. Our findings also have implications for the fields of social psychology and sociology, and point to future areas for interdisciplinary investigation and collaboration between these fields and STEM education. In the meantime, further research is needed to develop and improve measures of language-exclusive ideology and to identify and test strategies for disrupting such ideologies. Doing so could support broader efforts to advance racial and linguistic justice in STEM education and in education more broadly.
